# Sexual coercion of married women in Nepal

**DOI:** 10.1186/1472-6874-10-31

**Published:** 2010-10-28

**Authors:** Ramesh Adhikari, Jyotsna Tamang

**Affiliations:** 1 Geography and Population Department, Mahendra Ratna Campus Tribhuvan University, Kathmandu, Nepal; 2 Center for Research on Environment Health and Population Activities (CREHPA) Kathmandu, Nepal

## Abstract

**Background:**

Sexual coercion is an important public health issue due to its negative association with social and health outcomes. The paper aims to examine the prevalence of sexual coercion perpetrated by husbands on their wives in Nepal and to identify the characteristics associated with this phenomenon.

**Methods:**

The data used in this paper comes from a cross-sectional survey on "Domestic Violence in Nepal" carried out in 2009. A total of 1,536 married women were interviewed and associations between sexual coercion and the explanatory variables were assessed via bivariate analysis using Chi-square tests. Logistic regression was then applied to assess the net effect of several independent variables on sexual coercion.

**Results:**

Overall, about three in five women (58%) had experienced some form of sexual coercion by their husbands. Logistic regression analysis found that the literacy status of women, decision-making power regarding their own health care, husband-wife age differences, alcohol consumption by the husband, and male patriarchal control all had significant associations with women's experience of sexual coercion. Literate women had 28% less chance (adjusted odds ratio (aOR) = 0.72) of experiencing sexual coercion by their husbands than did illiterate women. Women who made decisions jointly with their husbands with regard to their own health care were 36% less likely (aOR = 0.64) to experience sexual coercion than those whose health care was decided upon by their mothers/fathers-in-law. On the other hand, women whose husbands were 5 or more years older than they were more likely to report sexual coercion (aOR = 1.33) than were their counterparts, as were women whose husbands consumed alcohol (aOR = 1.27). Furthermore, women who experienced higher levels of patriarchal control from their husbands were also more likely to experience sexual coercion by their husbands (aOR = 7.2) compared to those who did not face such control.

**Conclusion:**

The study indicates that sexual coercion among married women is widespread in Nepal. Programs should focus on education and women's empowerment to reduce sexual coercion and protect women's health and rights. Furthermore, campaigns against alcohol abuse and awareness programs targeting husbands should also focus attention on the issue of sexual coercion.

## Background

Sexual coercion may be defined as the act of forcing (or attempting to force) another individual through violence, threats, verbal insistence, deception, cultural expectations, or economic circumstances to engage in sexual behaviour against her/his will [1]. Worldwide, two of the most common forms of violence against women are physical and sexual abuse by their husbands or other intimate male partners. In many parts of the world, marriage is interpreted as granting men the right to unconditional sexual access to their wives and the power to enforce this access through force if necessary [2].

Many studies demonstrate that women are more vulnerable than men to sexual coercion [3-5]. An analysis of over 50 population-based surveys found that approximately 10-50% of adult women around the world reported having been physically assaulted by an intimate male partner (including their husbands) at some point in their lives [6]. Based on the self-reports of 24,000 women, the World Health Organization (WHO) reported that forced sex by intimate partners ranged from 4% in Serbia and Montenegro to 46% in Bangladesh and Ethiopia [7]. Representative studies conducted in the US and Australia revealed that around 20% of women have been sexually coerced [8-10]. Further research conducted among female factory workers in Nepal showed that 1 in 10 had been sexually coerced [11]. Similarly, a non-representative small scale study in Nepal [12,13] found that about half the husbands had forced their wives to have sex. A qualitative study conducted in 15 countries found that women had reported profoundly troubling experiences with regard to sex within marriage. These women frequently mentioned being physically forced to have sex and/or engage in types of sexual activity that they found degrading and humiliating [14]. Additional studies illustrate the long-term effects of coercion as young women who reported being coerced in their first sexual experience also had an increased chance of sexual violence in subsequent relationships [15,16].

Sexual coercion has profound emotional, psychological, social, physical, and health consequences both immediately and many years after the assaults [17,18]. Women who have been sexually coerced are significantly more likely to experience problems such as fear of intimacy, lack of sexual pleasure, and anxiety about sexual performance than are other women [9,19-21]. Sexually coerced women may feel powerless when it comes to insisting that their partner use a condom [22] and thus their partners may be less likely to use condoms [23]. Sexually coerced women have been found to be more likely to have unintended pregnancies [22,23] and abortions [24] and more likely to experience gynecological disorders such as irregular vaginal bleeding [25], vaginal discharge, painful menstruation [26,27], pelvic inflammatory disease [28], and sexual dysfunction [26,28,29] than other women. Coerced women are more likely to engage in risky sexual behaviour such as having sex with multiple partners [30], engaging in sex without a condom, and providing sex for money or drugs [8,9,19,31-33]. The prevalence of STI and HIV/AIDS has been found to be high among women who have faced violence [3,8,19]. Additionally, these women are more likely to smoke cigarettes and indulge in excessive alcohol consumption [9,22,34]. Generally speaking, sexually coerced women have poorer physical health [35], greater health anxiety [36,37] and poorer psychological health [9,19,22,23,38].

Since sexual coercion has negative impacts on psychological, physical, and sexual well-being, it is important to know the prevalence of sexual coercion and its determining factors. This paper aims to examine the prevalence in Nepal of sexual coercion perpetrated by husbands on their wives and to identify the characteristics associated with this phenomenon. Limited information about sexual coercion exists, and no representative study of sexual coercion experienced by married women has ever been conducted in Nepal.

## Methods

The data used in this paper were drawn from a cross-sectional survey on "Domestic Violence in Nepal" carried out in 2009. The survey was conducted with married women of reproductive age (15-49 years). One-on-one individual structured interviews were conducted in the survey and a two-staged stratified random sampling technique was employed for the selection of the respondents. Four districts (Achham, Gulmi, Rupendehi, and Ilam) out of Nepal's 75 districts were selected randomly with a total of 48 clusters (12 clusters times 4 districts). Among these districts, one district was from each of the Eastern and Far-Western regions, and two districts from the Western region. Education levels and socio-economic status of women in Gulmi, Rupendehi, and Ilam districts were similar to the national average, but women in Achham district had a lower socio-economic status. From each cluster, 32 households were chosen, and in each household one married woman was selected for the structured interview. In the case of there being more than one married woman in the same household, one was selected randomly. A total of 1,536 married women were interviewed.

Structured questionnaires were used for the data collection and included questions on socio-demographic characteristics of the woman and her spouse, along with her experience of domestic violence and sexual coercion by the husband [See additional file 1]. Pre-testing of the questionnaires with married women of reproductive age in non-sampled areas was carried out and the necessary changes in the questionnaire were incorporated.

Researchers were trained with regards to ethical and safety considerations while conducting research on these sensitive issues [39,40], and verbal informed consent was obtained from the participants before they enrolled in the study. The interview was conducted in a private space without the presence of a third party, and researchers requested that the interviewees not share any information with others in the study. The research team did not provide any financial compensation to the interviewed women; however, those women who needed counseling or treatment services were referred to the relevant sources of such assistance. Confidentiality of information was ensured by removing personal identifiers from the completed questionnaires, which protected respondents against adverse repercussions from participating in the study. The scientific review committee of the Social Inclusion Research Fund (SIRF)/SNV-Nepal reviewed and approved this study.

Sexual coercion experienced by a woman during her entire lifetime is used as the dependent variable in this study. Respondents were asked three questions:

i. Has your husband ever physically forced you to have sexual intercourse when you did not want to?

ii. Did you ever have sexual intercourse when you did not want to, because you were afraid of what your husband might do?

iii. Has your husband ever forced you to do something sexual that you found degrading or humiliating?

Sexual coercion was measured by analyzing the answers to the three questions (Cronbach's α = .71). Those women who reported experiencing at least one of the three forms of sexual coercion were considered to have experienced sexual coercion by a husband during their lifetime. Sexual coercion experienced during the lifetime of a woman will be stated as "sexual coercion" hereafter.

Similarly, some of the independent variables were also made into composite variables. For example, the variable "Women's perceived degree of power in relationships" was created by using the score from the following 6 questions (Cronbach's α = .67): i) whether a wife should obey her husband even if she disagrees, ii) whether a woman can choose her own friends even if her husband disapproves, iii) whether a woman has to have sex even when she does not want to, just to please her husband, iv) whether a man's opinion is more important than a woman's in important decision making within the relationship, v) whether it is important for a woman to give in to the man when they are arguing, and vi) whether a woman's husband will suspect her of being unfaithful if she asks him to use a condom. The score for these questions ranged from 0 to 6, though after making the composite index this variable was categorized into 3 levels-high power, medium power, and low power. Similarly, a composite index of male patriarchal control was made from the following 7 questions (Cronbach's α = .83): Would you say it is generally true that he: i) tries to keep you from seeing your friends, ii) tries to restrict contact with your family of birth, iii) insists on knowing where you are at all times, iv) ignores you and treats you indifferently, v) gets angry if you speak with another man, vi) is often suspicious that you are unfaithful, and vii) expects you to ask his permission before seeking health care for yourself. The composite variable range was from 0 to 7, and once again divided into 3 categories-no control, low control, and high control.

Both bivariate and multivariate techniques were applied to identify the factors associated with the likelihood of experiencing sexual coercion by a husband. The Chi-square test was used to test the association between the variables. The variables were then re-examined in multivariate analysis (binary logistic regression) in order to identify the significant associations. Before the multivariate analysis, multicollinearity between the variables was assessed and the highly correlated variables were removed from the logistic model. Statistical Package for the Social Sciences (SPSS v. 11.5) software was used for the analysis.

## Results

### Characteristics of the respondents

Two thirds of the 1,536 women were below the age of 34 years and about half of the women were literate. A large majority of women had 2 or more children and slightly more than a quarter of the women were in a "love marriage" (a union of two individuals based upon mutual love, affection, commitment, and attraction). About a third of respondents had husbands who were more than 5 years older than they were. Only about a fourth of women were involved in some kind of earning occupation, with a majority reporting that they were able to get financial or emotional support from their maternal family. A third of respondents mentioned that their fathers/mothers-in-law were the decision makers for their health care. As for their husbands, more than four-fifths were literate and almost two in five were involved in agricultural activities, while only a fifth were involved in service/business. More than half the women reported that their husbands consumed alcohol, and about two in five women reported that their husbands did not control them at all. Alternatively, nearly two in five women perceived that they had low power in the family (Table 1).

**Table 1:** Percentage distribution of women and experience of sexual coercion by a husband (N = 1536)

Characteristics	% of women	% who experienced sexual coercion	Total Number
**Age of women**		**ns**	
15-24	27.1	56.4	417
25-34	38.6	60.0	593
35+	34.2	58.0	526

**Literacy status of women**		***	
Illiterate	47.1	63.8	723
Literate	52.9	53.5	813

**Number of children ever born**		**ns**	
None/one	24.4	58.1	375
2-3 children	47.3	56.8	727
4 or more children	28.3	61.1	434

**Type of marriage**		**ns**	
Arranged marriage	71.2	57.0	1094
Love marriage	28.8	61.5	442

**Husband-wife age differences**		*	
No difference/older than husband	8.8	65.9	135
Husband is 1-5 years older	58.9	55.8	905
Husband is more than 5 years older	32.3	60.9	496

**Saving and credit group**		**ns**	
No involvement in the such group	49.8	59.9	765
Involvement in saving/credit group	50.2	56.8	771

**Women's earning**		*	
No involvement in earning	75.0	56.8	1152
Involvement in earning	25.0	63.0	384

**Support from maternal family**		**ns**	
No	17.4	61.4	267
Yes	82.6	57.7	1269

**Decision making for own health care**		***	
Fathers/Mothers-in-law	33.2	65.1	510
Respondents alone	45.9	57.4	705
Jointly with husband	20.9	49.5	321

**Education of husband**		***	
Illiterate	16.1	61.7	248
Primary education	29.8	65.9	458
Some secondary education and above	54.0	53.1	830

**Husband's occupation**		***	
Public/Business sectors	20.4	46.0	313
Agriculture	37.7	61.5	579
Daily wage/Labor	41.9	61.5	644

**Alcohol Consumption by husband**		***	
No	49.5	52.1	760
Yes	50.5	64.4	776

**Male patriarchal control**		***	
No control	37.6	42.9	578
Low control (1-2 issues)	46.3	61.2	711
High control (3-7 issues)	16.1	86.2	247

**Women's perceived degree of power in relationship**		***	
High power	16.0	51.0	245
Medium (1-3)	45.6	55.2	701
Low power (4-6)	38.4	65.1	590

**Total**	**100.0**	**58.3**	**1536**

Note: *** Chi-squared statistic significant at <0.001 ** = p < 0.01 * = p < 0.05, ns = not significant

### Forms of sexual coercion

Close to three in five women (58%) reported that their husbands had physically forced them to have sexual intercourse. Similarly, more than two in five (45%) mentioned that they had experienced unwanted sexual intercourse because they were afraid of what their husbands might do if they refused. A few women (3%) reported that their husband forced them to do something sexual that they found degrading or humiliating. Combining all three forms of sexual coercion shows that 58.3% (n = 896) out of 1,536 women had experienced at least one form of sexual coercion and that almost half (45%) had experienced at least two forms of sexual coercion (data not shown).

### Correlates of sexual coercion

Overall, about three in five women (58%) had experienced at least one of the three mentioned forms of sexual coercion by a husband. Table 1 shows a clear association between having experienced sexual coercion by a husband and other background variables such as women's literacy status, women's earnings, decision making with regard to women's own health care, husband-wife age differences, husband's education level, husband's occupation, alcohol consumption by the husband, male patriarchal control, and perception regarding power relations. For instance, a significantly higher (p < 0.001) proportion of illiterate women than literate women had experienced sexual coercion by a husband (64% vs. 54%). Also, a higher proportion of women (p < 0.05) who were the same age or older than their husbands or whose husbands were 5 years or older, had experienced sexual coercion compared to those who were 1 to 5 years younger than their husbands (Table 1).

Women involved in some form of earning occupation were found to have faced a significantly higher (p < 0.05) proportion of sexual coercion by their husbands than those with no employment. Furthermore, a significantly higher (p < 0.001) percentage of women who reported that the decision maker for their health care was their fathers/mothers-in-law had experienced sexual coercion by their husbands than did those who decided for themselves or jointly with their husbands (Table 1).

Husband's background characteristics also have a significant association with a woman's experience of sexual coercion. For instance, a significantly lower proportion of women whose husbands had a secondary or higher education had experienced sexual coercion compared to women whose husbands were illiterate. Similarly, a significantly lower percentage of women (p < 0.001) whose husbands' occupation was in the public or business sector had experienced sexual coercion compared to those whose husbands engaged in other occupations, such as agriculture or as daily wage labor. Moreover, a significantly higher (p < 0.001) proportion of women whose husbands consumed alcohol had experienced sexual coercion than did women whose husbands did not consume alcohol. Regarding perceptions towards power relations and male patriarchal control, women who perceived that they had less power faced more sexual coercion (p < 0.001), as did women whose husbands exerted more control over their everyday life (Table 1).

### Multivariate analysis

Logistic regression analysis was used to measure the strength of the association between various factors and the probabilities of women experiencing sexual coercion by their husbands. After assessing multicollinearity in the variables, it was found that "age of women" and "number of children ever born" were highly correlated (r = 0.63). Therefore the variable '"number of children ever born" was not entered in the logistic regression model.

As can be seen from Table 2, variables such as the literacy status of women, the decision making regarding one's own health, husband-wife age differences, the occupation of the husbands, alcohol consumption by the husband, and male patriarchal control were significant variables associated with having experienced sexual coercion by a husband. Literate women were 28% less likely (aOR = 0.72; 95% CI = 0.55-0.94) to experience sexual coercion by a husband than were illiterate women. Similarly, women who made decisions jointly with their husbands with regard to their own health care were 36% less likely to experience sexual coercion (aOR = 0.64; 95% CI 0.45-0.87) than were those women whose decisions were made by fathers/mothers-in-law. On the other hand, women whose husbands were working in agriculture or in the daily wage/labor sector were 56% (aOR = 1.56; 95% CI = 1.15-2.13) and 44% (aOR = 1.44; 95% CI = 1.05-1.97), respectively, more likely to experience sexual coercion compared to those women whose husbands worked in the public/business sectors. Furthermore, women whose husbands were older than themselves by 5 years or more had a 33% higher chance of experiencing sexual coercion (aOR = 1.33; 95% CI = 1.04-1.69) than did women whose husbands were only 1 to 5 years older. Women whose husbands consumed alcohol were 27% more likely (aOR = 1.27; 95% CI = 1.04-1.97) to report sexual coercion than were those whose husbands did not consume alcohol. Moreover, women who experienced higher levels of patriarchal control from their husband were more likely to face sexual coercion than were women who did not face such control. For instance, women who experienced low and high levels of patriarchal control were about twice (aOR = 1.99; 95% CI = 1.58-2.52) and 7 times (aOR = 7.24; 95% CI = 4.79-10.94), respectively, more likely to experience sexual coercion by a husband compared to those who had no male patriarchal control (Table 2).

**Table 2:** Adjusted odds ratios (aOR) and 95% confidence interval (CI) for having ever experienced sexual coercion by a husband

Variables	aOR	95% CI
**Age of women **15-24 (ref.)		
25-34	1.05	0.80-1.39
35+	0.86	0.63-1.17

**Literacy status of women **Illiterate (ref.)		
Literate	0.72*	0.55-0.94

**Type of marriage **Arranged marriage (ref.)		
Love marriage	1.26	0.96-1.64

**Husband-wife age differences **Husband is 1-5 years older (ref.)		
No difference/older than husband	1.41	0.94-2.12
Husband is more than 5 years older	1.33*	1.04-1.69

**Saving and credit group **No involvement in the such group (ref.)		
Involvement in saving/credit group	1.07	0.85-1.35

**Women's earning **No involvement in earning (ref.)		
Involvement in earning	1.24	0.95-1.62

**Support from maternal family **No (ref.)		
Yes	0.94	0.70-1.28

**Decision making for own health **Father/mother-in-law (ref.)		
Respondents alone	0.88	0.67-1.15
Jointly with husband	0.64**	0.45-0.87

**Education of husband **Illiterate (ref.)		
Primary	1.34	0.94-1.91
Some secondary and above	1.09	0.77-1.56

**Husband's occupation **Public/Business sectors (ref.)		
Agriculture	1.56**	1.15-2.13
Daily wage/Labor	1.44*	1.05-1.97

**Alcohol Consumption by husband **No (ref.)		
Yes	1.27*	1.04-1.97

**Male patriarchal control **No control (ref.)		
Low control (1-2 issues)	1.99***	1.58-2.52
High control (3-7 issues)	7.24***	4.79-10.94

**Women's perceived degree of power in relationships **High power (ref.)		
Medium(1-3)	1.08	0.78-1.49
Low power (4-6)	1.39	0.97-1.99

**-2 Log likelihood**	**1869.7**	

**Cox & Snell R Square**	**0.132**	

**Total N**	**1536**	

Note: *** = p < 0.001 ** = p < 0.01 * = p < 0.05

## Discussion

This study aimed to estimate the prevalence and examine the factors associated with sexual coercion in Nepal by husbands toward their wives. As in other parts of the world, sexual coercion is a serious problem in Nepal. The study shows that even with under-reporting on such a sensitive issue as sexual coercion, about three in five married women reported experiencing sexual coercion by their husbands, which is very high compared to studies conducted with Bangladeshi (46%), Ethiopian (46%) [7], American (20%-46%) [5,8,10], and Australian women (21%) [9].

Our study demonstrates that educated women were less likely to experience sexual coercion by a husband, a similar conclusion to a study conducted in India [41]. Our study also found that women who decided jointly about their own health care with their husbands were less likely to experience sexual coercion by their husbands compared to those whose decision making regarding their health care was made by fathers/mothers-in-law. One probable reason could be that the freedom these women had to decide jointly with their husband about their own health carried over to other matters, such as when to have sex, so the probability of their being coerced was less. Our study found that the greater the difference in age between husband and wife, the higher the chance of their being coerced by the husband. This could be due to the failure of older husbands and their younger wives to be able to share similar feelings and desires with respect to each other due to the large age gap. Although the odds of sexual coercion were high among women who were the same age or older than their husbands, this result was not significant and could be due to the small sample size in this category. These findings will require further investigation.

The occupation of the husband has a significant association with sexual coercion. Those women whose husbands worked in the public or private sectors such as business, government, and other professional positions were less likely to experience sexual coercion than were those whose husbands worked in agriculture or the labor sector. This result may be due to businessmen's, government workers', and professionals' work schedules and frequency of travel outside their home environment, which could make them more aware of their wives' sexual health and reproductive rights, though further investigation will be required.

Our study illustrates that respondents whose husbands consumed alcohol were more likely to experience sexual coercion than were those whose husbands did not consume alcohol. This finding is similar to other studies that have found alcohol to be a major cause of intimate partner violence [42-45].

The present study also found that women who were highly controlled by their husbands were more likely to face sexual coercion than were those without such controls. Feminists stress that gender and power structures in a patriarchal society are the root cause of intimate partner violence [46]. Studies conducted in the US found that the more patriarchal the norm is in a state, the higher the rate of intimate partner violence [46]. A study conducted in China also found that intimate partner violence is strongly associated with male patriarchal values [47].

There are some limitations in the interpretation of the results for this study. First, as pointed out previously, our sample is married women, so our results regarding the prevalence of sexual coercion should not be generalized to all women in Nepal. Second, because the cross-sectional design of the study and all of the items analyzed in the logistic regression analysis came from information at the time of survey, the analysis can only provide evidence of statistical association between those items and sexual coercion, and cannot show cause-effect relationships. Third, due to the sensitive nature of the issue, the prevalence of sexual coercion may have been under-reported. However, the privacy conditions surrounding the study and the good rapport that was built before the interview are likely to have minimized purposeful misreporting.

## Conclusion

Our study indicates that sexual coercion of married women by husbands is widespread and also a serious problem in Nepal, which could lead to devastating health problems for women. This study has found that educational status of respondents, decision making with regard to women's own health care, husband-wife age differences, husband's occupation, alcohol consumption by the husband, and male patriarchal control were the variables that had a statistically significant association with sexual coercion. Programs should focus on education for women and women's empowerment to reduce sexual coercion and to protect women's health and rights. In addition, campaigns against alcohol abuse and awareness programs targeting husbands should also cover the issue of sexual coercion.

## Competing interests

The authors declare that they have no competing interests.

## Authors' contributions

RA and JT conceived and designed the study. Both researchers were involved in data analysis, interpretation of the data, and drafted the manuscript. Both authors read and approved the final manuscript.

## Pre-publication history

The pre-publication history for this paper can be accessed here:

http://www.biomedcentral.com/1472-6874/10/31/prepub

## Supplementary Material

Additional file 1 **Questionnaire on the study entitled "Survey on Domestic Violence in Nepal"**. The questionnaire includes questions on socio-demographic characteristics of the woman and her spouse, her attitude regarding gender roles and her experience of domestic violence and sexual coercion by the husband. Click here for file
