# Supplementary material for: Sexual coercion of married women in Nepal
Source: BMC Womens Health. 2010 Oct 28;10:31. doi: 10.1186/1472-6874-10-31 (PMC2987890; doi:10.1186/1472-6874-10-31)
Supplement: Additional file 1 — Questionnaire on the study entitled "Survey on Domestic Violence in Nepal". The questionnaire includes questions on socio-demographic characteristics of the woman and her spouse, her attitude regarding gender roles and her experience of domestic violence and sexual coercion by the husband. [file 1472-6874-10-31-S1.DOC]

**ID no.**

**Domestic violence in Nepal**

**Social Inclusion Research Fund**

**2009**

**Informed consent**

Introduction

"Namaste”! My name is ..................... .

Purpose of the study

On behalf of the Social Inclusion Research Fund, we are here to conduct a study among women in order to find out their life experiences, relations between the partners and health.

Procedures

We are conducting the study in 4 districts with 1536 married of reproductive age. We have chosen you randomly. We would like to request you to participate in the study in order to obtain all these information.

Confidentiality

The information that we collect from this research study will be kept strictly confidential. Information about you that will be collected from the study will be stored in a file that will not have your name on it, but a number assigned to it instead. Only the principal investigator will have access to this file. The results of the study will be disseminated to the SIRF. But no information that identifies you to your name will disclosed.

During the interview there maybe many questions that is personal and sensitive. The information that you have given us will be kept confidential and will be used only for the study.

Voluntary participation

There will be no personal benefit in participating in this study. Participation in this study is voluntary. If you do not wish to answer any of the questions posed during the interview, you may say so and I will move on to the next question. Even after you agree to participate in the study, you will be free to leave the interview at any time you wish and/or to refuse to answer any question that you are uncomfortable with. If you choose not to talk to with us, it will not affect your access to services or information in anyway.

If you have any questions you may ask those now or later. If you wish to ask you questions later, you may contact Jyotsna Tamang, in the telephone number 5546487 or 5521717.

Shall I continue with the interview?

**Section 1: Introduction**

| **Q.N.** | **Questions** |
| --- | --- |
| 101 | District Name: |
| 102 | Name of VDC …………………………………………………….. |
| 103 | Cluster No. ______________________________________ |
| 104 | Ward No.: |
| 105 | Household No. |
| 106 | Name of household head_________________________ |
| 107 | Sex of head of the household: 1. Male  2. Female |
| 108 | Name of the interviewer_____________________ Date______________ |
| 109 | Name of field supervisor____________________ Date_____________ |

110 Household visit of respondent

| **Visited result** | Visited Date **Date Month Year** | **Result of visit** |
| --- | --- | --- |
| First Time |  |  |
| Second Time |  |  |
| Third Time |  |  |

**Visited result codes (code for 110)**

1. Interview completed 2. Interview incomplete 3. Respondent not at home

4. Respondent refused 5. Others (specify)...................

**Section 2: Household Questionnaire**

| SN | 201. Please give me the names of the persons who usually live in your household, starting with the head of the household. | 202. What is the relationship of (NAME) to the head of the household?* | 203. Is (NAME) male or female?  1. Male  2. Female | 204. Completed age in years | 205. Education of the person? (ask only those above 5 years)** | 206. Marital status (for those above age 10 years)?  1. Unmarried  2. Married  3. Widow/widower  4. Divorce/separated  5. Married but not gauna | 207. Has (NAME) ever been away from home for work or studies?  1. Yes  2. No | 208. Where?  1. Within district  2. Outside district  3. Outside country (specify) | 209. Eligible respondent (Currently married women aged 15-49 )  1. Eligible  2. Not eligible |
| --- | --- | --- | --- | --- | --- | --- | --- | --- | --- |
|  |  |  |  |  |  |  |  |  |  |
|  |  |  |  |  |  |  |  |  |  |
|  |  |  |  |  |  |  |  |  |  |
|  |  |  |  |  |  |  |  |  |  |
|  |  |  |  |  |  |  |  |  |  |
|  |  |  |  |  |  |  |  |  |  |
|  |  |  |  |  |  |  |  |  |  |
|  |  |  |  |  |  |  |  |  |  |
|  |  |  |  |  |  |  |  |  |  |
|  |  |  |  |  |  |  |  |  |  |

***Code for 202**

| 01=Head of household | 04=Daughter-in-law | 07= Parent-in-law | 10= Elder Brother or sister |
| --- | --- | --- | --- |
| 02=Wife or husband | 05= Grandchild | 08= Nephew, Niece | 11=Younger Brother or Sister |
| 03=Son or daughter | 06= Parent | 09=Co-wife |  |

****Code for 205**

| 01=Illiterate | 02=Non-formal education  03=Primary (1-5) | 04=Lower secondary (6-8)  05=Secondary (9-10) | 06=SLC and above |
| --- | --- | --- | --- |

210. Number of married women aged 15-49 years in the household……

211. Number of persons usually living in the household………………...

| **IF MORE THAN ONE ELIGIBLE WOMEN IN HH:**   - **RANDOMLY SELECT** ONE ELIGIBLE WOMAN FOR INTERVIEW. - **PUT CIRCLE AROUND LINE NUMBER OF WOMAN SELECTED.** ASK IF YOU CAN TALK WITH THE SELECTED WOMAN. IF SHE IS NOT AT HOME, AGREE ON DATE FOR RETURN VISIT.   **IF NO ELIGIBLE WOMAN IN HH:**   - **SAY “I cannot continue because I can only interview women 15–49 years old. Thank you for your assistance.”**   **FINISH HERE.** |
| --- |

# Section 3: Demographic and socio-economic background of the woman

| **Q.N.** | **Questions** | **Coding Categories** | **Code** | **Skip** |
| --- | --- | --- | --- | --- |
| 301 | How old were you at your last birthday? | Age in completed years.................... |  |  |
| 302 | Caste/ethnicity  Specify caste....................................... | Brahmin/Chhetri…….............  Magar/Gurung/Rai/Limbu/TamangDalit (Kami/Damai/Sarki).............  Terai Brahmin/Chhetri..................  Terai Janajati................................  Terai Dalit....................................  Terai Non Dalit...........................  Muslim........................................ | 1  2  3  4  5  6  7  8 |  |
| 303 | What is the highest level of education that you achieved? | Number of years…  NFE…………………………….. | 99 |  |
| 304 | Where did you grow up? | This community/neighbourhood… Another VDC…………….……….  Another district…………………...  Another country…………………. | 1  2  3  4 |  |
| 305 | How long have you been living continuously in this (Name of community)? | Number of years .....  Less than 1 year…………………  Lived all her life ………………… | 97  98 |  |
| 306 | Do any of your family of birth live close enough by that you can easily see/visit them? | They live nearby……………….  They don’t live nearby………….  Living with family of birth …….  Don’t know…………………… | 1  2  3  8 |  |
| 307 | How often do you see or talk to a member of your family of birth?  Would you say at least once a week, once a month, once a year, or never? | At least once a week…………….  At least once a month…………..  At least once a year ……………  Never (Hardly ever)……………  Other (specify) ........................  Don't know/don’t remember…… | 1  2  3  4  88 |  |
| 308 | When you need help or have a problem, can you usually count on members of your family of birth for support? | Yes………………………..………  No……………………..…………  Don’t know/don’t remember……. | 1  2  8 |  |
| 309 | Do you have any close friends to who you can rely on in case you need help or have a problem? | Yes………….……………….  No…………………………....  Don’t know…………………. | 1  2  8 |  |
| 310 | What is your religion? | No religion…………………..  Hindu………………………..  Buddhist……………………..  Christian……………………..  Muslim………………………  Other (specify) ........................ | 1  2  3  4  5 |  |
| 311 | What are the **main** sources of income in your family? | Agriculture……………….  Business………………………  Craftsmanship………………  Govt. service………………...  Daily wages…………………..  Others (Specific)………….. | 1  2  3  4  5 |  |
| 312 | Do you attend a group, organization or association?  IF YES:  What kind of group, organization or association?  IF NO, PROMPT:  Organizations like women’s or community groups, or political associations. | Yes………….……………….  No………………………….... | 1  2 |  |
|  | Civic/political | 1 2 |  |  |
|  | Economic/savings and credit group | 1 2 |  |  |
|  | Women’s organization | 1 2 |  |  |
|  | Mother’s group……………….. | 1 2 |  |  |
|  | Others (specify)…………….. | 1 |  |  |
| 313 | Do you save at the saving and credit group?  When you save at the saving and credit group, whose money do you **usually** save? | Not participated in saving and credit group/does not save………  My own........................................  My husband's..............................  My father-in-law's....................  My mother-in-law's....................  Others (specify)…………….. | 1  2  3  4  5 | 315 |
| 314 | Who decides how the money from the savings and credit group is to be used? | Self…………………………..  Husband……………………..  Both self and husband……….  Father-in-law…………………  Mother-in-law……………….  Other (specify)……………… | 1  2  3  4  5 |  |
| 315 | Has anyone ever prevented you from attending a meeting or participating in an organization?  IF YES, ASK  Who prevented you?  *Multiple response possible* | Not prevented……………….  Husband……………………..  Parents……………………….  Parents-in-law………………..  Others (specify)…………….. | 1  2  3  4 |  |
| 316 | Do you consume alcohol? | Yes………….……………….  No………………………….... | 1  2 | 401 |
| 317 | How often do you drink alcohol?  Would you say every day or nearly every day, once or twice a week, 1-3 times a month, occasionally less than once a month, never? | Every day…………………….  Nearly every day…………….  Once or twice a week………..  1-3 times a month……………  Occasionally, less than once a month………………………..  Never………………………… | 1  2  3  4  5  6 |  |

**Section 4: Marriage, Fertility and Contraception**

Now I would like to talk about your marriage and your children.

| **Q.N.** | **Questions** | **Coding Categories** | **Code** | **Skip** |
| --- | --- | --- | --- | --- |
| 401 | Before your marriage, did you know about sexual intercourse that generally takes place with spouse? | Yes………….……………….  No…………………………....  Don’t know…………………. | 1  2  8 |  |
| 402 | How old were you when you got (first) married? | Age…………. |  |  |
| 403 | Was your marriage (first) arranged or was it a love marriage? | Arranged marriage…………...  Love marriage……………….. | 1  2 |  |
| 404 | At what age you started living with your (first) husband (Age at *Gauna* in *Terai*)? | Age………….. |  |  |
| 405 | Have you ever been pregnant? | Yes……………………………  No……………………………  Don't know………………… | 1  2  8 | 417 |
| 406 | How many times have been pregnant so far? | Number of times. |  |  |
| 407 | Have you ever given birth? | Yes……………………………  No…………………………….  First time pregnant………….. | 1  2  3 | 409 |
| 408 | How many living children do you have? | No. of sons……….  No. of daughters… … |  |  |
| 409 | When you ever became pregnant, did you want to become pregnant then, did you want to wait until later, did you not want any (more) children at all? | Become pregnant then.............  Wait until later.........................  Not want children.....................  Don’t know……………………  Refused/no answer................... | 1  2  3  8  9 | 412  412 |
| 410 | How many times did such pregnancies occur? | Number……….. |  |  |
| 411 | I would like to ask you about your last pregnancy.  At the time you became pregnant with this child (NAME), did you want to become pregnant then, did you want to wait until later, did you not want any (more) children at all? | Become pregnant then.............  Wait until later.........................  Not want children.....................  Don’t know……………………  Refused/no answer................... | 1  2  3  8  9 |  |
| 412 | When you were last pregnant, did your husband want you to become pregnant then, wanted to wait until later, did not want any (more) children at all? | Become pregnant then..............  Wait until later.........................  Not want children.....................  Don’t know…………………..  Refused/no answer................... | 1  2  3  8  9 |  |
| 413 | When you were last pregnant, did you see anyone for an antenatal check?  IF YES: Whom did you see?  Anyone else? | No one....................................  Doctor.....................................  Obstetrician/gynaecologist.....  Nurse/midwife........................  Auxiliary nurse.......................  Traditional birth attendant......  Others (specify)…………….. | 1  2  3  4  5  6 |  |
| 414 | Did your husband stop you, encourage you, or have no interest in whether you received antenatal care for your **last** pregnancy? | Stop.........................................  Encourage...............................  No interest..............................  Don’t know/don’t remember...  Refused/no answer................... | 1  2  3  8  9 |  |
| 415 | Did your other family members stop you, encourage you, or have no interest in whether you received antenatal care for your **last** pregnancy? | Stop.........................................  Encourage...............................  No interest..............................  Does not live with family.......  Don’t know/don’t remember...  Refused/no answer................... | 1  2  3  7  8  9 |  |
| 416 | When you were last pregnant, did your husband/partner have preference for a son, a daughter or did it not matter to him whether it was a boy or a girl? | Son...........................................  Daughter.................................  Did not matter........................  Don’t know/don’t remember....  Refused/no answer.................. | 1  2  3  8  9 |  |
| 417 | Have you or your husband ever used anything, or tried in any way, to delay or avoid getting pregnant? | Yes…………………………..  No……………………………  Never had intercourse……….  Don’t know/don’t remember…  Refused/no answer………….. | 1  2  3  8  9 | 412 |
| 418 | Are you or your husband **currently** doing something, or using any method, to delay or avoid getting pregnant? | Yes…………………………..  No……………………………  Don’t know/don’t remember…  Refused/no answer………….. | 1  2  8  9 | 421 |
| 419 | What (main) method are you currently using?  *If more than one, only mark main method* | Pills…………………………..  Injectables……………………  Implants (Norplant)…………..  IUD………………………….  Calendar method……………..  Female sterilization…………  Condoms…………………….  Male sterilization……………  Withdrawal…………………..  Others (specify)…………  Don’t know/don’t remember…  Refused/no answer…………... | 1  2  3  4  5  6  7  8  9  88  99 | 421 |
| 420 | Does your (current) husband know that you are using a method of family planning? | Yes…………………………..  No……………………………  Don’t know/don’t remember…  Refused/no answer………….. | 1  2  8  9 |  |
| 421 | Has your husband ever refused to use a method or tried to stop you from using a method to avoid getting pregnant? | Yes…………………………..  No……………………………  Don’t know/don’t remember…  Refused/no answer………….. | 1  2  8  9 | 501 |
| 422 | In what ways did he let you know that he disapproved of using methods to avoid getting pregnant?  *Multiple response possible* | Told me he did not approve…  Shouted/got angry……………  Threatened to beat me……….  Threatened to leave/ throw me out of home……………………….  Beat me/physically assaulted….  Took or destroyed method….  Others (specify)………….. | 1  2  3  4  5  6 |  |

# Section 5: Husband’s background

Now I would like to ask you a few things about your husband.

| **Q.N.** | **Questions** | **Coding Categories** | **Code** | Skip |
| --- | --- | --- | --- | --- |
| 501 | What is the age of your husband? | Completed age in years | |  |
| 502 | What is the highest level of education that your husband has completed? | Number of years. |  |  |
| 503 | What is the occupation of your husband? | Agriculture………………...  Daily wages …………………  Teacher………………………  Army…………………………  Police……………………….  Business……………………..  Others (Specify)…………….. | 1  2  3  4  5  6 |  |

| 504 | Is your husband living with you? | Yes………….……………….  No………………………….... | 1  2 | 506 |
| --- | --- | --- | --- | --- |
| 505 | If not, how often does your husband come to visit you? | Usually……………………….  Sometime…………………….  Rare………………………... | 1  2  3 |  |
| 506 | Does/did your husband have any other wives while being married (having a relationship) with you? | Yes…………………………..  No…………………………..  Don’t know………………….  Refused/no answer………….. | 1  2  8  9 | 509 |
| 507 | How many wives does/did he have (including yourself)? | Number of wives……..  Don’t know………………….  Refused/no answer………….. | 8  9 |  |
| 508 | Are/ were you the first, second....Wife? | Position…......................  Don’t know………………….  Refused/no answer………….. | 8  9 |  |
| 509 | Did your marriage involve dowry/bride price payment? | Yes …….…………………..  No …………………………..  Don’t know/don’t remember…  Refused/no answer………….. | 1  2  3  8 | 511 |
| 510 | Overall, do you think that the amount of dowry payment has had a positive impact on how you are treated by your husband and his family, a negative impact, or no particular impact? | Positive impact………………  Negative impact……………..  No impact……………………  Don’t know/don’t remember…  Refused/no answer…………… | 1  2  3  8  9 |  |
| 511 | Do you think that your husband drinks alcohol? | Yes………….……………….  No…………………………....  Don’t know…………………. | 1  2  8 | 513 |
| 512 | How often does (did) he drink alcohol?  Would you say Every day, Nearly every day, Once or twice a week, 1-3 times a month, Occasionally, less than once a month, Never | Every day…………………….  Nearly every day…………….  Once or twice a week………..  1-3 times a month……………  Occasionally, less than once a month………………………..  Never…………………………  Don’t know………………….. | 1  2  3  4  5  6  8 |  |
| 513 | Does he (did) take any drugs that are not prescribed by the doctor? | Yes………….……………….  No……………………………. | 1  2 |  |

**Section 6: Attitudes on Gender and power**

| **Q.N.** | **Questions** | **Coding** | **Code** | | | | | | **Skip** |
| --- | --- | --- | --- | --- | --- | --- | --- | --- | --- |
| 601 | **In your opinion……** | **Yes** | **No** | **DK** | | | | |  |
|  | 1. Household work should only be done by women? | 1 | 2 | 8 | | | | |  |
|  | 2. Family planning is only women’s issue? | 1 | 2 | 8 | | | | |  |
| 602 | **In your opinion……** | **Yes** | **No** | **DK** | | | | |  |
|  | 1. Should a wife obey her husband even if she disagrees? | 1 | 2 | 8 | | | | |  |
|  | 2. Is it important for a man to show his wife/partner who is the boss? | 1 | 2 | 8 | | | | |  |
|  | 3. If a man wants to have sex and a woman doesn’t then does she need to have sex to please him? | 1 | 2 | 8 | | | | |  |
|  | 4. Is it ok if others outside of the family intervenes when a man mistreats his wife? | 1 | 2 | 8 | | | | |  |
|  | 5. Is a man’s opinion more important than woman’s in important decision making in a relationship? | 1 | 2 | 8 | | | | |  |
|  | 6. If a man and woman are arguing, is it important for her to give in so that they stop arguing? | 1 | 2 | 8 | | | | |  |
|  | 7. If you asked your husband to use condom would he think you are having sex with someone else? | 1 | 2 | 8 | | | | |  |
| 603 | Do you think that male sex is superior to the female? Complementary or inferior? | Superior……………………..  Complementary……………..  Inferior………………………. | | | | | 1  2  3 | |  |
| 604 | Why do you think so?  ………………………………………………………………….………..  ………………………………………………………………….……….. | | | |  | | |  |  |
| 605 | Before taking any important decision, do you think about what the society will say? | Yes………………………….  No…………………………… | | | | 1  2 | | |  |
| 606 | In your opinion, does a man have a good reason to hit his wife if: | **Yes** | **No** | **DK** | | | | |  |
|  | 1. She does not complete her household work to his satisfaction | 1 | 2 | 8 | | | | |  |
|  | 1. She disobeys him | 1 | 2 | 8 | | | | |  |
|  | 1. She neglects the children | 1 | 2 | 8 | | | | |  |
|  | 1. She refuses to have sexual relations with him | 1 | 2 | 8 | | | | |  |
|  | 1. She asks him whether he has other girlfriends |  |  |  | | | | |  |
|  | f) He suspects that she is unfaithful | 1 | 2 | 8 | | | | |  |
| 607 | In your opinion, can a married woman refuse to have sex with her husband if: | **Yes** | **No** | **DK** | | | | |  |
|  | 1. She doesn’t want to | 1 | 2 | 8 | | | | |  |
|  | 1. He is drunk | 1 | 2 | 8 | | | | |  |
|  | 1. She is sick | 1 | 2 | 8 | | | | |  |
|  | 1. He mistreats her | 1 | 2 | 8 | | | | |  |
|  | e) She knows that her husband has sex with another woman | 1 | 2 | 8 | | | | |  |

**Section 7: Women’s experiences**

When two people marry or live together, they usually share both good and bad moments. I would now like to ask you some questions about your current and past relationships and how your husband treats (treated) you. If anyone interrupts us I will change the topic of conversation. I would again like to assure you that your answers will be kept secret, and that you do not have to answer any questions that you do not want to. May I continue?

| 701 | In general, do (did) you and your (current or most recent) husband discuss the following topics together: | **Yes** | **No** | **DK** | |  |
| --- | --- | --- | --- | --- | --- | --- |
|  | 1. Things that have happened to him in the day | 1 | 2 | 8 | |  |
|  | 1. Things that happen to you during the day | 1 | 2 | 8 | |  |
|  | 1. Your worries or feelings | 1 | 2 | 8 | |  |
|  | 1. His worries or feelings | 1 | 2 | 8 | |  |
| 702 | Have you discussed about contraception in the last 12 months? | Yes………….…………………No………………………….......  Don’t know………………… | | | 1  2  8 |  |
| 703 | Have you ever heard of a woman who has experienced physical violence from her husband in this community? | Yes………….……………….  No…………………………....  Don’t know…………………. | | | 1  2  8 | 705 |
| 704 | Why do you think that such violence occurs?  ………………………………………………….  …………………………………………………. |  | | |  |  |
| 705 | In your relationship with your (current or most recent) husband, how often would you say that you quarrelled?  Would you say rarely, sometimes or often? | Rarely 1  Sometimes 2  Often 3  Don’t know/don’t remember 8  Refused/no answer..................... | | | 1  2  3  8  9 |  |
| 706 | I am now going to ask you about some situations that are true for many women. Thinking about your (current or most recent) husband, would you say it is generally true that he: | **Yes** | **No** | **DK** | |  |
|  | 1. Tries to keep you from seeing your friends | 1 | 2 | 8 | |  |
|  | 1. Tries to restrict contact with your family of birth | 1 | 2 | 8 | |  |
|  | 1. Insists on knowing where you are at all times | 1 | 2 | 8 | |  |
|  | 1. Ignores you and treats you indifferently | 1 | 2 | 8 | |  |
|  | 1. Gets angry if you speak with another man |  |  |  | |  |
|  | 1. Is often suspicious that you are unfaithful |  |  |  | |  |
|  | 1. Expects you to ask his permission before seeking health care for yourself | 1 | 2 | 8 | |  |

| 707 | The next questions are about things that happen to many women, and that your current husband may have done to you.  Has your current husband ever…. | A) (**If YES continue with B.  If NO skip to next item**)  YES NO | | B) Has this happened in the past 12 months?  (**If YES ask C only. If NO ask D only**)  YES NO | | C) In the past 12 months would you say that this has happened once, a few times or many times? (**after answering C, go to next item**)  One Few Many | | |
| --- | --- | --- | --- | --- | --- | --- | --- | --- |
|  | 1. Insulted you or made you feel bad about yourself? | 1 | 2 | 1 | 2 | 1 | 2 | 3 |
|  | 1. Belittled or humiliated you in front of other people? | 1 | 2 | 1 | 2 | 1 | 2 | 3 |
|  | 1. Done things to scare or intimidate you on purpose (e.g. by the way he looked at you, by yelling and smashing things)? | 1 | 2 | 1 | 2 | 1 | 2 | 3 |
|  | 1. Threatened to hurt you? | 1 | 2 | 1 | 2 | 1 | 2 | 3 |
| 708 | Has he ever…. | A)  (**If YES continue with B.  If NO skip to next item**)  YES NO | | B)  Has this happened in the past 12 months?  (**If YES ask C only. If NO ask D only**)  YES NO | | C)  In the past 12 months would you say that this has happened once, a few times or many times? (**after answering C, go to next item**)  One Few Many | | |
|  | 1. Slapped you or thrown something at you that could hurt you? | 1 | 2 | 1 | 2 | 1 | 2 | 3 |
|  | 1. Pushed you or shoved you or pulled your hair? | 1 | 2 | 1 | 2 | 1 | 2 | 3 |
|  | 1. Hit you with his fist or with something else that could hurt you? | 1 | 2 | 1 | 2 | 1 | 2 | 3 |
|  | 1. Kicked you, dragged you or beaten you up? | 1 | 2 | 1 | 2 | 1 | 2 | 3 |
|  | 1. Choked or burnt you on purpose? | 1 | 2 | 1 | 2 | 1 | 2 | 3 |
|  | 1. Threatened to use or actually used a gun, knife or other weapon against you? | 1 | 2 | 1 | 2 | 1 | 2 | 3 |

| 709 |  | A)  (**If YES continue with B.  If NO skip to next item**)  YES NO | | | B)  Has this happened in the past 12 months?  (**If YES ask C only. If NO ask D only**)  YES NO | | C)  In the past 12 months would you say that this has happened once, a few times or many times? (**after answering C, go to next item**)  One Few Many | | | | |
| --- | --- | --- | --- | --- | --- | --- | --- | --- | --- | --- | --- |
|  | 1. Did your husband ever physically force you to have sexual intercourse when you did not want to? | 1 | | 2 | 1 | 2 | 1 | | 2 | | 3 |
|  | 1. Did you ever have sexual intercourse you did not want to because you were afraid of what your husband might do? | 1 | | 2 | 1 | 2 | 1 | | 2 | | 3 |
|  | 1. Did your husband ever forced you to do something sexual that you found degrading or humiliating? | 1 | | 2 | 1 | 2 | 1 | | 2 | | 3 |
| 710 | Has you husband’s family ever physically beaten you? | | Yes………….…………………No…………………………....... | | | | | 1  2 | | 713 | |
| 711 | Who did this? | | Mother-in-law………………..  Father-in-law…………………  Sister-in-law………………….  Others (specify)………… | | | | | 1  2  3 | |  | |
| 712 | Has this happened in the last 12 months? | | Yes………….…………………No…………………………....... | | | | | 1  2 | |  | |
| 713 | You said that you have been pregnant (TOTAL) times. Was there ever a time when you were slapped, hit or beaten by your husband while you were pregnant? | | Yes……………………….  No…………………………..  Never been pregnant………….  Don’t know/don’t remember…  Refused/no answer…………… | | | | | 1  2  7  8  9 | | 719 | |
| 714 | Did this happen in one pregnancy, or more than one pregnancy? In how many pregnancies were you beaten? | | Number of pregnancies beaten…… | | | | | | |  | |
| 715 | Did this happen in the last pregnancy? | | Yes………………………….  No…………………………..  Don’t know/don’t remember…  Refused/no answer…………… | | | | | 1  2  8  9 | |  | |
| 716 | Were you ever punched or kicked in the abdomen while you were pregnant? | | Yes…………………………….  No……………………………..  Don’t know/don’t remember…  Refused/no answer…………… | | | | | 1  2  8  9 | |  | |
| 717 | Did you face any problems with your pregnancy or child birth due to such violence? | | Yes…………………………….  No…………………………..  Don’t know/don’t remember…  Refused/no answer…………… | | | | | 1  2  8  9 | | 719 | |
| 718 | What problem did you face? | | Heavy bleeding……………….  Abdomen pain…………………  Miscarriage………………….  Still birth………………………  Others (specify)………. | | | | | 1  2  3  4 | |  | |

| 719 | When you were small did your father ever beat your mother? | Yes……………………………  No…………………………….  They did not live together……..  Don’t know/don’t remember…  Refused/no answer…………… | 1  2  3  8  9 |  |
| --- | --- | --- | --- | --- |
| 720 | Has your father-in-law ever beaten your mother-in-law? | Yes……………………………  No…………………………….  They did not live together……..  Don’t know/don’t remember…  Refused/no answer…………… | 1  2  3  8  9 |  |
| **721** | **NOTE: Verify whether answered YES to any question on physical violence,  see question 708** | **Yes, physical violence ……...**  **No physical violence ……….** | **1**  **2** |  |
| **722** | **NOTE: Verify whether answered yes to any question on sexual violence,  see question 709** | **Yes, sexual violence ………...**  **No, sexual violence …………..** | **1**  **2** |  |

**Section 8: Injuries**

Now I would like to talk a little about your health.

| **Q.N.** | **Questions** | | **Coding** | | | | **Code** | | **Skip** |
| --- | --- | --- | --- | --- | --- | --- | --- | --- | --- |
| 801 | I would now like to ask a few questions about your health  In general, would you describe your overall health as excellent, good, fair, poor or very poor? | | Excellent……………………………  Good……………………………….  Fair…………………………………  Poor…………………………………  Very poor……………………………  Don’t know/don’t remember………..  Refused/no answer…………………. | | | | 1  2  3  4  5  8  9 | |  |
| **802** | **NOTE: REFER TO QUESTION 708 & 709 TO CODE** | | **Has faced physical/sexual violence…**  **Not faced physical/sexual violence….** | | | | **1**  **2** | | **901** |
|  | I would now like to learn more about the injuries that you experienced from (any of) your husband’s acts that we have talked about (MAY NEED TO REFER TO SPECIFIC ACTS RESPONDENT MENTIONED IN SECTION 8). By injury, I mean any form of physical harm, including cuts, sprains, burns, broken bones or broken teeth, or other things like this. | | | | | | | |  |
| 803 | Have you ever been injured as a result of these acts by (any of) your husband/partner(s)? | | Yes………………………………….  No…………………………………..  Don’t know/don’t remember………  Refused/no answer………………… | | | | 1  2  8  9 | | 808 |
| 804 | What type of injury or health problem did you have?  Please mention any injury due to (any of) your husbands acts, no matter how long ago it happened.  MARK ALL  PROBE:  Any other injury? | Cuts, punctures, bites………………….  Scratch, abrasion, bruises…………….  Sprains, dislocations…………………  Burns…………………………………  Penetrating injury, deep cuts, gashes…  Broken eardrum, eye injuries………….  Fractures, broken bones………………..  Broken teeth……………………………  Internal injuries………………………  Other (specify): ……………… | | Yes  1  1  1  1  1  1  1  1  1 | Has this happened in the past 12 months?  YES NO DK | | | |  |
|  |  |  |  |  | 1  1  1  1  1  1  1  1  1 | 2  2  2  2  2  2  2  2  2 | | 8  8  8  8  8  8  8  8  8 |  |

| 805 | In your life, did you ever receive health care for this injury (these injuries)? Would you say, sometimes or always or never? | Yes, sometimes……………………..  Yes, always…………………………  No, never…………………………….  Don’t know/don’t remember………..  Refused/no answer…………………. | 1  2  3  8  9 | 808 |
| --- | --- | --- | --- | --- |
| 806 | Did you tell a health worker the real cause of your injury? | Yes………………………………….  No…………………………………..  Don’t know/don’t remember………  Refused/no answer………………… | 1  2  3  8  9 | 808 |
| 807 | If yes, what did the health provider suggest?  ………………………………………………..  ……………………………………………….. | |  |  |
| 808 | Have you ever experienced any psychological problems as a result of these violence (fear, tension, depression etc)? | Yes……………………………  No…………………………… | 1  2 | 810 |
| 809 | What psychological problems did you face? | Fear…………………………….  Tension………………………..  Depression……………………..  Suicidal feeling……………….  Others (Specify)………….. | 1  2  3  4 |  |
| 810 | Have you ever tried to take your life? | Yes……………………………….  No………………………………….  Don’t know/don’t remember……….  Refused/no answer………………… | 1  2  8  9 |  |
| 811 | You told me earlier that you had (Refer to question 410) number of unintended pregnancies.  Would you say that any of such pregnancies had occurred because of your husband had used force to have sexual intercourse? | Yes……………………………………  No…………………………..…………  Never had unintended pregnancy……..  Never experienced sexual violence…..  Can't say…………..………………. | 1  2  3  4  8 | 901 |
| 812 | When was the last time such pregnancies occurred due to your husband’s act? | 0-3 months ago.............……...  3-6 months..................………..  6-9 months ago................……  9-12 months ago..........………....  1-2 years ago...........……….....  2-3 years ago............……….....  3-4 years ago...........……….....  4 years ago............……….... | 01  02  03  04  05  06  07  08 |  |
| 813 | What happened to that pregnancy? | Currently pregnant…………………  Live birth…………………. ………  Tried to abort but did not succeed...  Aborted.......................……….....  Miscarried......................………  Still birth……………………… | 1  2  3  4  5  6 |  |

**Section 9: Impact and coping**

I would now like to ask you some questions about what effects your husband’s acts have had on you. With acts I mean… (REFER TO SPECIFIC ACTS THE RESPONDENT HAS MENTIONED IN SECTION 8).

| **Q.N.** | **Questions** | **Coding** | **Code** | **Skip** |
| --- | --- | --- | --- | --- |
| 901 | Note: Check with Q.721 and 722 and circle the code | Experienced sexual and  physical violence……………………  Never experienced sexual and physical violence………………….. | 1  2 | 1001 |
| 902 | Are there any particular situations that tend to lead to your husband/partner’s behaviour?  *Refer to acts of physical violence mentioned before.*  PROBE: Any other situation?  *Multiple responses possible* | No particular reason………………..  When man drunk…………………..  Money problems…………………..  Difficulties at his work…………….  When he is unemployed……………  No food at home……………………  Problems with his or her family…….  She is pregnant………………………  He is jealous of her………………….  She refuses sex……………………..  She is disobedient…………………..  Others (Specify)……………..…….. | 1  2  3  4  5  6  7  8  9  10  11 |  |
| 903 | Have you ever done anything to avoid the violence that you face? | Yes……………………………  No…………………………… | 1  2 | 905 |
| 904 | What did you do?  *Multiple responses possible* | Fought with him…………………..  Went to the neighbour’s house……  Went to maternal house…………..  Shouted………………………….  Others (Specify)……………..…….. | 1  2  3  4 |  |
| 905 | During the times that you were hit, did you ever fight back physically or to defend yourself?  IF YES: How often? Would you say once or twice, several times or most of the time? | Never………………………………..  Once or twice……………………….  Some times………………………….  Several times………………………..  Don’t know/don’t remember………..  Refused/no answer…………………. | 1  2  3  4  8  9 | 907 |
| 906 | What was the effect of you fighting back on the violence at the time?  Would you say, that it had no effect, the violence became worse, the violence became less, or that the violence stopped, at least for the moment. | No change/no effect…………………  Violence became worse…………….  Violence became less……………….  Violence stopped……………………  Don’t know/don’t remember………...  Refused/no answer………………….. | 1  2  3  4  8  9 |  |
| 907 | In what way, if any, has your husband/partner’s behaviour (the violence) disrupted your work or other income-generating activities?  *Multiple responses possible* | Work not disrupted………………….  Partner interrupted work……………  Unable to concentrate……………….  Unable to work/sick leave…………..  Lost confidence in own ability………  Others (Specify)………….. | 1  2  3  4  5  6 |  |

| 908 | Who have you told about his behaviour?  *Multiple responses possible*  PROBE: Anyone else? | No one………………………………  Friends……………………………..  Parents………………………………  Brother or sister…………………….  Uncle or aunt………………………..  Husband’s family………………….  Children…………………………….  Neighbours…………………………..  Police……………………………….  Doctor/health worker………………..  Priest…………………………………  Counsellor…………………………...  NGO/women’s organization…………  Local leader………………………….  Others (Specify)………….. | 1  2  3  4  5  6  7  8  9  10  11  12  13  14 | 910 |
| --- | --- | --- | --- | --- |
| 909 | Did anyone ever try to help you?  IF YES, Who helped you?  *Multiple responses possible*  PROBE: Anyone else? | No one……………………………..  Friends…………………………….  Parents………………………………  Brother or sister…………………….  Uncle or aunt………………………..  Husband’s family…………………..  Children……………………………..  Neighbours………………………….  Police……………………………….  Doctor/health worker……………….  Priest………………………………..  Counsellor…………………………..  NGO/women’s organization……….  Local leader………………………..  Others (Specify)………….. | 1  2  3  4  5  6  7  8  9  10  11  12  13  14 | 1001 |
| 910 | What were the reasons that you did not go to any of these?  *Multiple responses possible* | Don’t know/no answer………………  Fear of threats/consequences/  More violence……………………….  Violence normal/not serious………..  Embarrassed/ashamed/afraid would not be believed or would be blamed..  Believed not help/know other women not helped……………………………  Afraid would end relationship……….  Afraid would lose children………….  Bring bad name to family…………..  Others (Specify)………….. | 1  2  3  4  5  6  7  8 |  |

**Section 10: Autonomy**

Now I would like to ask you some questions about things that you own and your earnings. We need this information to understand the financial position of women nowadays. In relation to this I would also like to talk about some situations that are true for many women.

| **Q.N.** | **Questions** | **Coding** | **Code** | | **Skip** |
| --- | --- | --- | --- | --- | --- |
| 1001 | Do you have any of your own personal belongings? | Yes…………………………  No…………………………… | | 1  2 | 1003 |
| 1002 | What do you own? | Land/House…………………  Jewellery……………………  Money………………………  Livestock……………………..  Other (specify) ........................ | |  |  |
| 1003 | Do you earn something to contribute to the family income? If yes, what is the nature of your job? | Do not earn/housewife.............  Teacher.....................................  Daily wages labourer................  Govt service holder..................  Private service holder………  Petty business……..…..…….  Other (specify) ........................ | | 1  2  3  4  5  6 | 1007 |
| 1004 | How much do you earn per month? |  | |  |  |
| 1005 | Who mainly decides how the money you earn will be used? | Self…………………………..  Husband……………………..  Both self and husband……….  Father in law…………………  Mother-in-law……………….  Other (specify)……………… | | 1  2  3  4  5 |  |
| 1006 | Are you able to spend the money you earn how you want yourself, or do you have to give all or part of the money to your husband? | Self/own choice……………..  Give part to husband ……….  Give all to husband…………  Other (specify)………………  Don’t know…………………. | | 1  2  3  88 |  |
| 1007 | How is your financial status compared to that of your neighbours? | Higher status than neighbour  The same……………………  Lower status…………………  Don’t know………………… | | 1  2  3  8 |  |
| 1008 | Have you ever given up/refused a job for money because your husband did not want you to work? | Yes………………………  No……………………………  Don’t know/don’t remember…  Refused/no answer………… | | 1  2  8  9 |  |
| 1009 | Has your husband ever taken your earnings or savings from you against your will?  IF YES: Has he done this once or twice, several times or many times? | Never………………………..  Once or twice………….…….  Several times………….…….  Many times/all of the time…  N/a (does not have savings/earnings)………….…  Don’t know/don’t remember…  Refused/no answer…………. | | 1  2  3  4  5  8  9 |  |
| 1010 | Does your husband ever refuse to give you money for household expenses, even when he has money for other things?  IF YES: Has he done this once or twice, several times or many times? | Never……………………..  Once or twice…………….  Several times……………….  Many times/all of the time….  N/a (does not have savings/earnings)……………..  Don’t know/don’t remember…  Refused/no answer…………. | | 1  2  3  4  5  8  9 |  |
| 1011 | Who is the most dominant partner in your relationship? | Always me…………………..  Mostly me……………………  Both of us…………………..  Mostly partner………………  Always partner……………… | | 1  2  3  4  5 |  |
| 1012 | Now I would like to ask decision-making process of your house. Generally who give the decision on the following matter ? | 1. Respondent 2. Husband 3. Both husband and wife 4. Mother-in-law 5.Father-in-law 6. Other (specify)…… | |  |  |
|  | 1. Purchase of domestic goods | 1 2 3 4 5 6 | |  |  |
|  | 2. Visit to family members/relative/friends | 1 2 3 4 5 6 | |  |  |
|  | 3. Caring of own health | 1 2 3 4 5 6 | |  |  |
|  | 4. Given birth or not | 1 2 3 4 5 6 | |  |  |
|  | 5. No of desire children | 1 2 3 4 5 6 | |  |  |
|  | 6. Using or not using of FP method | 1 2 3 4 5 6 | |  |  |
|  | 7. When to have sex | 1 2 3 4 5 6 | |  |  |

**Section 11: Household Amenities**

| **Q.N.** | **Questions** | **Coding** | **Code** | **Skip** |
| --- | --- | --- | --- | --- |
| 1101 | Does your household have: | **Yes No** |  |  |
|  | Electricity…………..…..…. | 1 2 |  |  |
|  | A radio………………….…. | 1 2 |  |  |
|  | A television………………… | 1 2 |  |  |
|  | Telephone…………….……. | 1 2 |  |  |
|  | A gas stove………….……… | 1 2 |  |  |
|  | A kerosene stove………….... | 1 2 |  |  |
|  | Private tub well or tap…….… | 1 2 |  |  |
| 1102 | Does any member of your household own: | **Yes No** | **NA** |  |
|  | A bicycle……………….. | 1 2 | 3 |  |
|  | A motorbike……………. | 1 2 | 3 |  |
|  | A tractor………………… | 1 2 | 3 |  |
| 1103 | Type of floor of the house  *(Please observe)* | Clay………….……...…… PCC……..………………… Tile……………..………….  Other (specify)…………… | 1  2  3 |  |
| 1104 | Type of roof (main material) of the house  *(Please observe)* | Tile………………………….  Thatched…………………….  Wood………………………  Stone………………………..  Tin…………………………..  Cement/brick……………….  Other (specify)………….. | 1  2  3  4  5  6  7 |  |
| 1105 | How many rooms in your household are used for sleeping? | Number….. |  |  |

**Section 12: Exposure to mass media**

| **Q.N.** | **Questions** | **Coding** | **Code** | **Skip** |
| --- | --- | --- | --- | --- |
| 1201 | How often do you read newspapers and magazines? | Almost everyday…………….  Sometimes (2-3 days a week).  Rarely………………………..  Don’t read…………………..  Not applicable………………. | 1  2  3  4  5 |  |
| 1202 | How often do you listen to the radio? | Almost everyday……….…….  Sometimes (2-3 days a week)..  Rarely………………………...  Don’t listen…………………..  Not applicable……………..… | 1  2  3  4  5 |  |
| 1203 | How often do you view television? | Almost everyday……………..  Sometimes (2-3 days a week)..  Rarely………………………  Don’t watch…………………  Not applicable………………. | 1  2  3  4  5 |  |

Thank you very much for your time. This is the end of the interview.
